# Triage practices for emergency care delivery: a qualitative study among febrile patients and healthcare workers in a tertiary care hospital in Nepal

**DOI:** 10.1186/s12913-024-10663-3

**Published:** 2024-02-08

**Authors:** Bipin Adhikari, Lava Shrestha, Manjita Bajracharya, Nishika Aryal, Anuja Rajbhandari, Ramesh K. Maharjan, Santa K. Das, Jyotshna Sapkota, Kevin K. A. Tetteh, Debashish Das

**Affiliations:** 1 Institute of Medicine, Kathmandu, Nepal; 2 Mahidol-Oxford Tropical Medicine Research Unit, Faculty of Tropical Medicine, Mahidol University, Bangkok, Thailand; 3 Centre for Tropical Medicine and Global Health, University of Oxford, Oxford, UK; 4 Emergency Medicine, Tribhuvan University Teaching Hospital, Kathmandu, Nepal; 5 FIND, Campus Biotech, Chemin des Mines 9, 1202 Geneva, Switzerland

**Keywords:** Triage, Emergency department, Patient journey, In-depth interview

## Abstract

**Background:**

Improving screening and triage practices is essential for early severity assessments at the first point of contact and ensuring timely attention by healthcare workers (HCWs). The main objective of this study was to explore the triage process among febrile patients and HCWs in the emergency department (ED) of a tertiary care hospital in a resource-constrained setting.

**Methods:**

This qualitative study was conducted from March to May 2023 at the ED of Tribhuvan University Teaching Hospital (TUTH), Nepal. The study included in-depth interviews with febrile patients (*n* = 15) and HCWs (*n* = 15). Additionally, direct observation notes (*n* = 20) were collected to document the triage process and patients’ experiences in the ED. Data underwent thematic analysis using the Interpretative Phenomenological Analysis (IPA) approach.

**Results:**

The ED of TUTH offered comprehensive triage services with clear delineation for the severity of febrile patients in line with the World Health Organization (WHO) guidelines. Nonetheless, challenges and constraints were identified. In the ED, evenings were generally the busiest period, and the triage process was not thorough during night shifts. Perception of triage was limited among patients and variable among HCWs. Digitalizing recordings of patient information including payment was deemed necessary for effective management of patients’ waiting times at the triage station. High patient throughput added pressure on HCWs and had a potential influence on the delivery of services. Availability of medical equipment and space were also identified as challenges, with patients sometimes compelled to share beds. There were constraints related to waste disposal, hygiene, cleanliness, and the availability and maintenance of washrooms. Febrile patients experienced delays in receiving timely consultations and laboratory investigation reports, which affected their rapid diagnosis and discharge; nonetheless, patients were satisfied with the overall healthcare services received in the ED.

**Conclusions:**

Improving current triage management requires resource organization, including optimizing the waiting time of patients through a digitalized system. Urgent priorities involve upgrading visitor facilities, patient consultations, laboratory investigations, hygiene, and sanitation. HCWs’ recommendations to resource the ED with more equipment, space, and beds and a dedicated triage officer to ensure 24-hour service, together with training and incentives, warrant further attention.

**Supplementary Information:**

The online version contains supplementary material available at 10.1186/s12913-024-10663-3.

## Background

Emergency care, also known as emergency medicine, is the medical specialty concerned with the care and treatment of acutely ill or injured patients who need immediate medical attention. The practice of emergency medicine includes the initial evaluation, diagnosis, treatment, and coordination of care among multiple clinical specialties [1]. The objective of emergency care is to stabilize patients, prevent their condition from worsening, and initiate the necessary treatments as early as possible to improve outcomes [2]. Evidence suggests that longer boarding times in the emergency department (ED) i.e., time spent in the ED before transfer to inpatient units, are associated with higher mortality risks [3].

Triage is an integral part of emergency care as it is essential to assign limited resources to unlimited medical needs. The main purpose of ED triage is to identify severely ill patients for prompt management allocating priorities to patients according to severity. An effective triage thus ensures that the patient receives the level and quality of care appropriate to clinical needs and that the available resources are well utilized [4]. Clinical efficiency emphasizes that the patient receives medical care appropriate to their needs on time. The concept of urgency or timeliness is central to triage in emergency medicine. Triage systems facilitate systematic assessment and treatment, patient comfort and reassurance, documentation of patient needs, and communication with patients and caregivers regarding the nature of their problems and the process of medical care [5]. Not all patients presenting to the ED require the same level of urgent treatment, attention, and resource allocation. In addition, the growing demand for emergency healthcare, access block, and ED overcrowding add to the need for better allocation of resources [6].

Emergency care in low- and middle-income countries (LMICs) is often precarious because of constraints in resources (e.g. health human resources, infrastructure and equipment) compounded by high volumes of patients attendance often beyond the threshold of health care services. Nonetheless, emergency care in LMICs also holds immense potential for making significant improvements on the health outcomes of critically ill patients [7]. But little is known about the ED triage process in resource-constrained settings [8, 9]. Existing triage tools and algorithms are often developed and validated in high-income settings, which may not be directly applicable and feasible in resource-limited contexts. There is a need for a better understanding of ED triage systems in LMICs taking into account local healthcare capacity, and available resources. The main objectives of this study were to investigate the triage process among febrile patients and healthcare workers (HCWs) in the ED of a tertiary care hospital in a resource-constrained setting, identify challenges and opportunities for improvement in current triage practices, and gain insights into patients’ ED journey.

## Methods

### Design and overview

This was an exploratory, qualitative study that utilized observations, interviews, and discussions among patients, caregivers, and HCWs from March to May 2023 in the ED of Tribhuvan University Teaching Hospital (TUTH), Kathmandu, Nepal. Two experienced female qualitative researchers, NA and AR, with a Bachelor of Public Health and prior experience in qualitative research, were briefed on the study context, objectives, and methodology. Following the orientation, BA (MD, DPhil), an expert social scientist trained the researchers on methodological approaches and procedures for conducting observations and in-depth interviews (IDIs) using the relevant interview guide (Additional File 1). The interview guides were prepared by BA, DD (PhD), and LS (MD) to respond to the research questions. IDIs were piloted among the study team (LS and BA) and family members and patients’ caregivers to ensure the comprehensibility of the guide and thematic relevance. Specifically, tailored to the study, the triage process was explained to the qualitative researchers, and they were asked to conduct pilot observations under the supervision of the social scientist at the outset. The first few observation notes were reviewed by the study investigators (BA and DD) and adaptations were made to optimize their utility, for instance observing and making informal interactions with the respondents. The outcome from these observation notes informed the existing triage process, challenges (constraints), and areas for further improvements.

### Data collection

During the direct observation of the triage process, the researchers (NA and AR) had conversations with patients and HCWs to expand on the observation notes, also referred to as a process of ‘shadowing’. To ensure that the notes accurately captured the events and the process, the researchers liaised and communicated with the HCWs regularly. A total of 20 observation notes were collated which captured and documented the diverse context (e.g., busiest period, resources, and infrastructure within the ED). The workload of HCWs and their coordination with each other at the ED was also explored.

Fifteen IDIs of HCWs were undertaken which included nine staff nurses/triage nurses (one among them was a nurse in charge), four medical officers (MOs), one specialist in emergency medicine with a doctorate in medicine (DM), and one health assistant (Table 1). The interviews focused on exploring the resources at hand, such as guidelines and specialized training, that are utilized for the triage process. It aimed to identify the systematic approach taken during triage, while also examining the challenges and potential opportunities associated with carrying out triage effectively.

**Table 1:** Socio-demographic characteristics of the participants: A) Febrile patients, B) Healthcare workers (HCWs)

Identification Number	Age category (yr)	Sex	Qualification	District	Referral case
A) Febrile patients					
Pt_1	20–30	M	Higher Secondary	Kathmandu	No
Pt_2	30–40	F	Higher Secondary	Gorkha	Yes
Pt_3	50–60	F	Can’t read or write	Tanahun	Yes
Pt_4	30–40	M	Lower Secondary	Nuwakot	Yes
Pt_5	50–60	M	Can’t read or write	Kathmandu	No
Pt_6	30–40	F	Higher Secondary	Rautahat	Yes
Pt_7	70–80	F	Can’t read or write	Gorkha	Yes
Pt_8	20–30	F	Undergraduate	Kathmandu	No
Pt_9	10–20	M	Higher Secondary	Rautahat	Yes
Pt_10	50–60	M	Informal Education	Birtamode	No
Pt_11	10–20	M	Higher Secondary	Khotang	Yes
Pt_12	20–30	F	Undergraduate	Kathmandu	No
Pt_13	40–50	F	Higher Secondary	Kathmandu	No
Pt_14	20–30	M	Undergraduate	Kathmandu	No
Pt_15	50–60	F	Lower Secondary	Butwal	Yes
Identification Number	Age category (yr)	Sex	Qualification	Position	Work experience (yr)
B) Healthcare workers (HCWs)					
Cl_1	20–30	F	BNS	Staff Nurse	2
Cl_2	30–40	F	BSc Nursing	Staff Nurse	6
Cl_3	30–40	F	BSc Nursing	Staff Nurse	10
Cl_4	20–30	F	MBBS	MO	0.1
Cl_5	20–30	F	MBBS	MO	0.5
Cl_6	30–40	M	DM	DM	0.4
Cl_7	40–50	F	BNS	Nurse In-Charge	19
Cl_8	20–30	M	HA	HA	0.2
Cl_9	40–50	F	BNS	Staff Nurse	15
Cl_10	20–30	F	BSc Nursing	Staff Nurse	2
Cl_11	20–30	F	BNS	Staff Nurse	2
Cl_12	20–30	M	MBBS	MO	0.5
Cl_13	30–40	F	BNS	Staff Nurse	13
Cl_14	30–40	F	BSc Nursing	Staff Nurse	6
Cl_15	20–30	F	MBBS	MO	0.6

*BNS* Bachelor of Nursing Science, *BSc* Bachelor of Science, *Cl* Clinician, *DM* Doctor of Medicine, *F* female, *HA* Health Assistant, *M* male, *MBBS* Bachelor of Medicine and Bachelor of Surgery, *MO* Medical Officer, *Pt* Patient, *yr* year

The researchers were trained on a qualitative approach to document ‘Patient Journey Mapping’ which was recorded through the IDIs with only febrile patients attending the ED. Febrile patients were purposively selected in this study based on the epidemiological burden at the ED of TUTH. The patients were approached after inquiring with HCWs about the patients presenting with fevers. When the HCWs were occupied, the febrile patients were identified by reviewing patients’ medical records. Interviewers were specifically recruited for this study, so the topic of research was novel for them. All respondents were approached face-to-face at the ED. Except for one patient who could not continue the interview because of the persistence of cough, none of the approached respondents refused to participate.

The febrile patients and researchers had no prior interactions. The interview covered three phases: pre-visit, visit, and post-visit, with a focus on investigating the factors that influenced patients’ treatment-seeking behaviour. It also explored patients’ views regarding the services they received during their visits to the ED, including issues such as waiting times, the screening or triage process, and the strengths and weaknesses of the available services. Lastly, the interview gathered insights from patients about their perspectives after they visited the ED. A total of 15 febrile patients from the ED of green, yellow, red, and observation areas were enrolled in the study. Febrile patients who were below 18 years of age, were not physically and mentally fit and who were unwilling to offer time for conversations were all excluded. To ensure adequate diversity, the IDIs with febrile patients were conducted based on their severity of illnesses, geographic region, gender, ethnicity, and background (Table 1). Except for patients in the red area, respondents did not have their caretakers with them during the interviews. All interviews were conducted face-to-face in Nepali and were audio-recorded, translated, and transcribed into English. Any incomplete clues and implications while sharing preliminary results among specific respondents were further explored through discussions and interactions but none of the transcripts were shared for comments or corrections [10].

### Data analysis

Participant responses in interviews were anonymized, and any identifying information was removed from the analyzed transcripts. The transcripts and observation notes were collated into Microsoft Word and Excel spreadsheets which were analyzed using Interpretative Phenomenological Analysis (IPA) by two coders (NA and AR) followed by critical reflection on it by BA [11]. Through an examination of the excerpts, in addition to existing themes from the interview guide, a set of emerging themes were identified, which were further discussed among investigators. This qualitative study has been reported following the Consolidated Criteria for Reporting Qualitative Research (COREQ) guidelines (Additional File 2) [12].

## Results

### Overview

The triage process at the ED of TUTH followed a triage guideline set by the World Health Organization (WHO). Emergency rooms were allocated to different areas with a specific number of beds available, the equipment provided, and the assignment of HCWs in each area. The study identified potential challenges and opportunities in the current triage practice. Challenges included substantial information gaps on triage and its guidelines, the lack of a digital record and payment system, and the low HCW-to-patient ratio which may hinder effective service delivery. Additional challenges echoed by both patients and HCWs included the lack of resources, poor hygiene and cleanliness, problems with the dispatch system, and lack of proper facilities for patient parties. The challenges identified in this study highlight areas for further improvement.

### Emergency department setting

The ED comprised several designated areas, including the triage, green, yellow, and red areas respectively, and an observation room. In the triage area, there was usually one triage nurse available to assess patients and make their tickets, which typically took approximately five minutes to complete. The green area had 13 beds including one room for the Orthopedic department, and two beds for procedures, while the yellow and red areas had 14 and 8 beds, respectively. Each room was equipped with counters to accommodate HCWs and assist the patients. Additionally, a monitor was available at the triage counter for monitoring the patient’s vital signs (respiratory rate, oxygen saturation, pulse, blood pressure, temperature). The ED also had a dedicated section for medicines and essential medical equipment (e.g., tracheostomy tube, and ambu bag/manual resuscitator). The existing payment process in the ED involved a manual cash payment for emergency tickets, lab tests, and procedures. During the peak patient flow, individuals accompanying the patients were compelled to wait in line for about 15–20 minutes to complete the cash payment procedure.

In case of emergencies, patients were directly escorted to the red area with their tickets either by the triage staff themselves or by the security guard. Once they had arrived in the red area, the patient was handed over to the HCW, and the nurse was instructed to fill out the patient’s ticket. In each section of the ED, there were two nurses and two medical doctors assigned, along with two health assistants in the green area and 1 DM in the red area. However, during the night shift, there was a reduced number of HCWs compared to the day and evening shifts.

### Red area

Each bed in the red area was equipped with a monitor; eight in total to record the patient’s vital signs continuously. However, the availability of ventilators was limited, with only 2 units present, one of which was portable. In addition, there were two electrocardiogram (ECG) machines and a portable ultrasonography (USG) machine available. There were four infusion pumps and four syringe pumps. Each bed contained suction, oxygen supplies, and curtains were provided when needed to ensure the privacy of patients.

### Yellow area

Beside the counter of the yellow area, there were waste disposal dustbins in three different colours: red, yellow, and green. Several monitors, including oxygen pumps, were placed near the beds. The necessary equipment, such as pulse oximeters was readily available. Adequate arrangements were made for the storage of medicines and essential tools. However, it was noted that patients in both the red and yellow areas had to utilize the same restroom facilities.

### Green area

There were two to three chairs available for the visitors in the green area. In addition, there was another procedure room which was also referred to as the “green area”. The green room was usually crowded, as it served as the primary entrance leading to other sections of the facility. During the night shift, all patients were initially directed to the green counter upon their arrival. As required, various equipment such as ECG machines, trolleys, or wheelchairs were made available to patients who needed them.

### Observation area

Each of the 23 beds in the facility was equipped with electrical outlets and lockers for patients’ belongings. A total of seven monitors with one oxygen cylinder and one ECG machine were available in the observation room. Some beds included chairs for patients’ visitors, and a washing basin was placed just across the entrance of the room. To ensure the privacy of patients, curtains were provided when needed. A designated section was allocated for essential medicines and tools. Near the reception area, two dustbins were present. However, there was no particular colour coding for waste disposal categories. Therefore, all types of waste were disposed of in those two dustbins; except for syringes or needles, which were disposed of at the medicine trolley. There were no separate toilets for males and females.

### Triage process

The triage process is a routine part of emergency care. The colour-coded guideline of triage, placed at the entrance of the ED, was properly followed by HCWs while conducting the triage process. However, no subsequent training specifically focused on triage was provided.

*“The triage system started at first around the year 2068 BS* [verified date is 2070 BS or 2013 AD]*, they provided us with a pamphlet, brought from Australia which included five categories of the triage system. After that no other guidelines have been provided, it is all done through what we studied and what we practiced.”*

Cl_9, 40–50 years, female.

While the HCWs have participated in other training programs, they have acquired fragmented knowledge about triage through those training sessions.

*“I haven’t had any training regarding triage, but I got training for emergency nursing care for three months which included the triage process as well. I took the training about 2-3 years ago. It was conducted by Nursing and Service Education, there were 13 participants in the training*.”

Cl_3, 30–40 years, female.

The triage system at TUTH adopted WHO triage categories, which were displayed on a board at the entrance of the ED setting. The first category, labelled red, represented the highest priority level. The red area was assigned to patients with life-threatening conditions that required immediate attention, such as cardiac arrest, stroke, ongoing seizure, and respiratory distress. The second category, assigned as yellow, was for patients with moderate conditions. They required medical evaluation and treatment within a reasonable timeframe. Examples of patients assigned as yellow included those with fever with signs of lethargy, moderate shortness of breath, and oxygen saturation > 90–95%. The third category, labelled as green was applied to patients with minor or non-urgent conditions. These patients could wait for medical attention as their conditions were not immediately life-threatening, e.g., minor wounds, and minor lacerations requiring suturing. The HCWs demonstrated a clear understanding of the concept of triage and acknowledged its significance. The triage process was effectively carried out by dedicated staff stationed at the triage counter between 8 AM and 8 PM. However, during the night shift, the triage process was relaxed, and lacked a functional system, leading to patients being assessed by the MOs in the green area and allocated directly from the green area to the designated room. Nonetheless, the majority of HCWs emphasized the necessity of implementing a 24-hour triage system in the ED to ensure high-quality service.

Moreover, one of the most noteworthy aspects of ED was its re-triaging practice, which allowed patients to be relocated to different areas based on the severity of their condition when necessary. Re-triaging was carried out according to the condition of the patient by the MOs based on their clinical judgement during the ward round. Most HCWs were aware that patients often required re-triaging and notified the MO who would subsequently transfer the patients to relevant departments.

Most of the HCWs reported that the ED lacked sufficient human resources, beds, and space. They noted that having one member of triage staff assigned to the triage area was insufficient and recommended at least two triage staff members at the triage counter to ensure immediate assessment of the patients and minimize waiting time. Even though there were occasionally perceptual conflicts, the HCWs were found to be professional and well-coordinated.

*“When the patient flow is high in the yellow zone, the staff of the green zone goes to the yellow zone and helps them.”*

Cl_8, 20–30 years, male.

The HCWs worked in three shifts: morning (9 AM-3 PM), evening (3 PM–9 PM), and night (9 PM-9 AM). The evening period was identified as the busiest time in the ED, compared to the morning period which was relatively less crowded.

*“The Out-Patient Department session usually starts around 11.30 AM [9 am] and when it finishes at around 4 PM, the flow of patients gets high in the ED.”*

Cl_7, 40–50 years, female.

Furthermore, there was a higher influx of patients during the summer season compared to the winter season. During times of disaster or any major events of mass casualties, the HCWs received prior notification from higher authorities. In addition, HCWs from various wards were generally assembled to assist in the ED.

*“When any mass casualties occur, there is a siren used as a signal. When that siren is played, staff from all the departments come in one place. This is the protocol, to do the preparation and stay. There needs to be a person with experience who can guide the team properly.”*

Cl_12, 20–30 years, male.

**Fig. 1:**
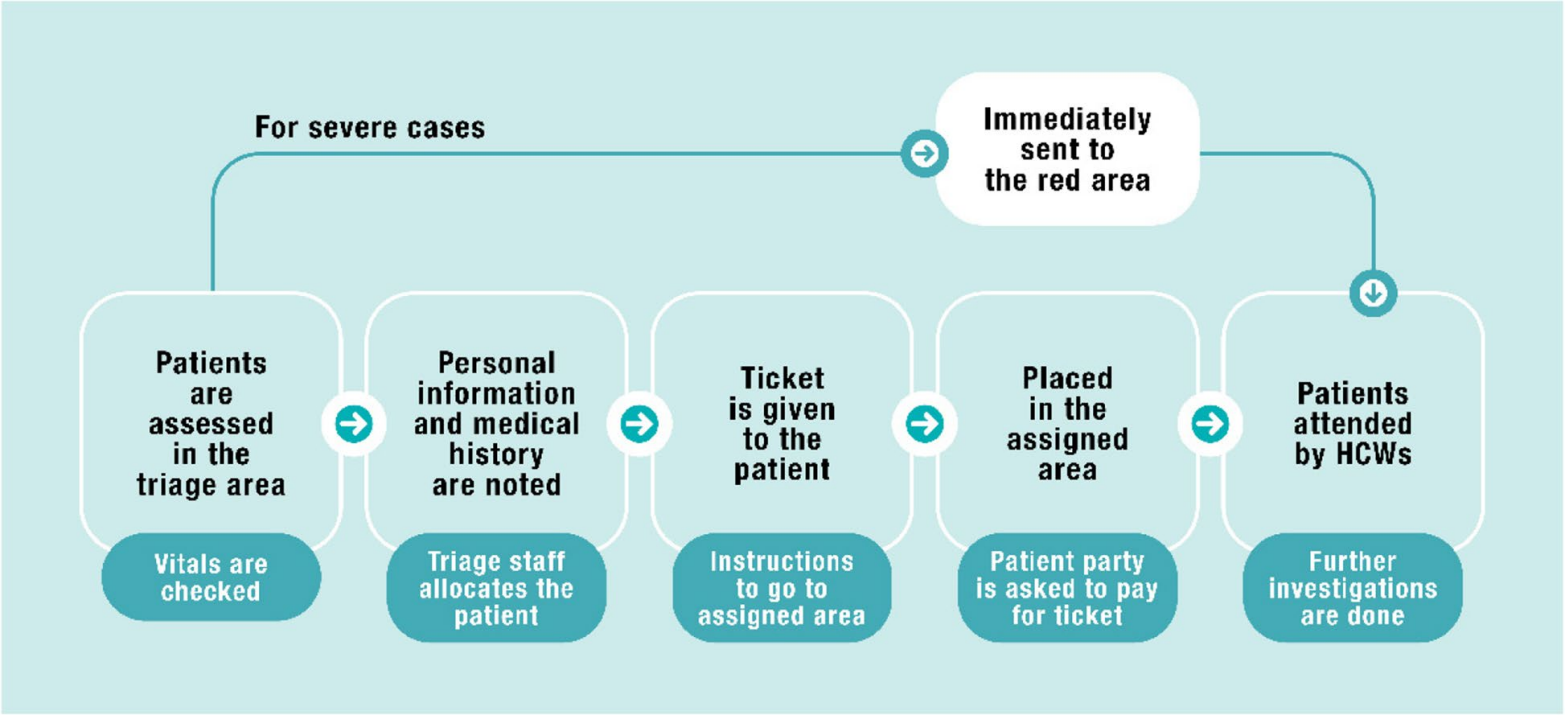
Triage process of febrile patients in the ED

This flowchart (Fig. 1) illustrates the sequence of the febrile patients’ triage process upon entering the ED. Initially, the patients were attended by a triage nurse stationed at the triage counter and then assigned to their designated ED room. Once allocated, medical doctors and nurses provided further care and assistance to the patients. Critically ill patients (severe cases) requiring immediate medical attention were promptly directed to the red area to ensure timely treatment without any delay, considering the severity and the potentially life-threatening nature of their condition.

**Fig. 2:**
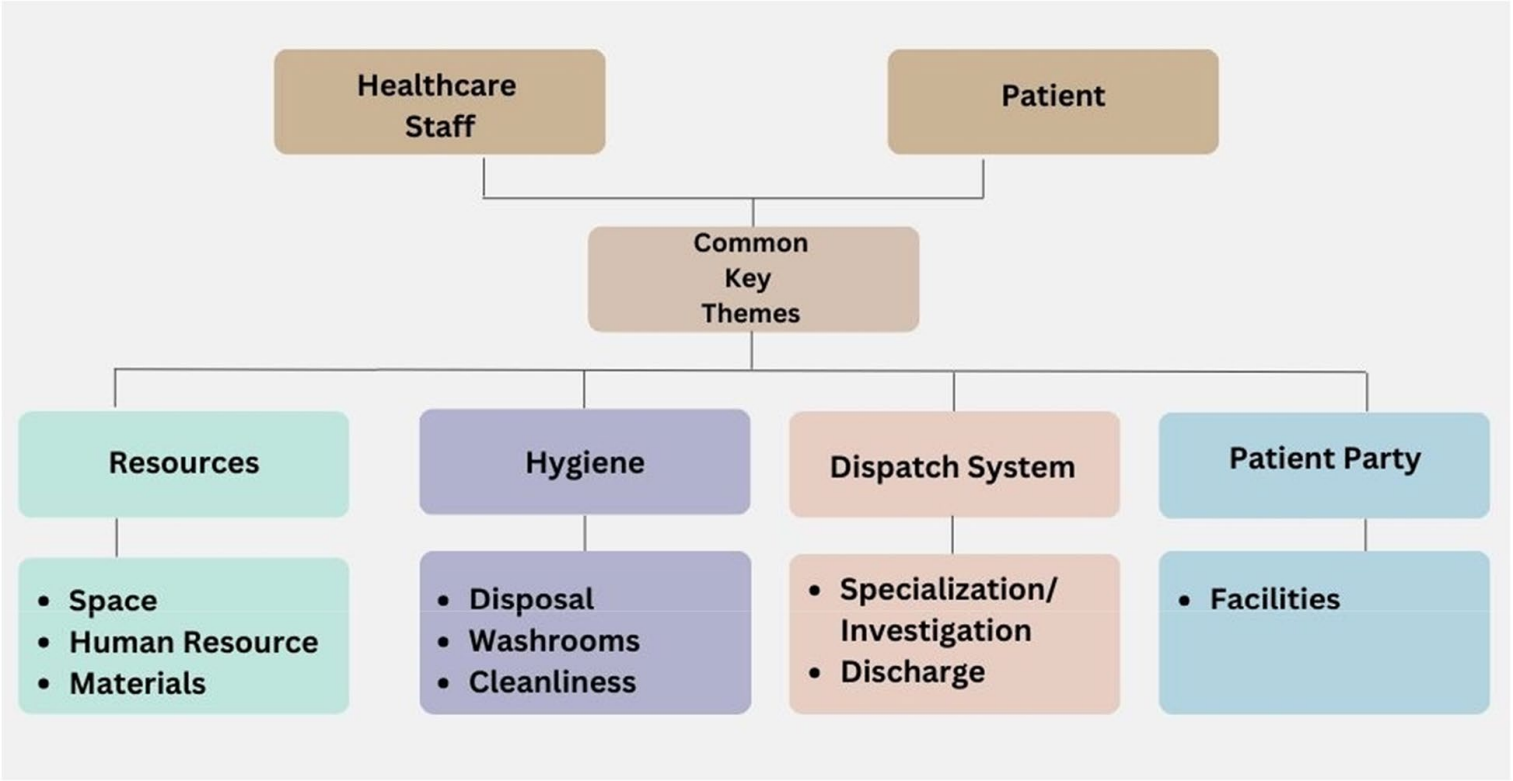
Common themes revolve around patients and healthcare workers

Figure 2 represents the primary focus of the discussion, which revolves around recurring themes related to patients and HCWs.

### Pre-visit experiences affecting treatment-seeking behavior

Febrile patients primarily chose to visit the hospital because it offered affordable healthcare services. Many of these patients had been suffering from prolonged fever and enduring pain before deciding to seek treatment at ED. The decision to visit the ED or hospital was typically made by male members of the household, such as partners, fathers, brothers, or sons, and the individuals seeking treatment did not hesitate in their decision.

*“We have been coming here since my father’s period. My father always visited this hospital for his checkup. So, we know this hospital that’s why we came here. We all decided to come here for the checkup. My family always comes here if anyone gets sick, we like the service provided here. It is cheaper than other private hospitals.”*

Pt_2, 30–40 years, female.

*“My brothers brought me here. My brother-in-law said that the treatment service in the Teaching Hospital is nice, so I came here.”*

Pt_9, 10–20 years, male.

However, it was worth noting that one participant initially intended to attend a different hospital but was denied admission and thus ended up in this hospital. A substantial number of participants were referred to the hospital and were specifically advised to visit the hospital.

*“I was referred to this hospital from Trishuli Hospital, Nuwakot.”*

Pt_4, 30–40 years, male.

The TUTH hospital was well-known and recognized by both patients and their accompanying parties. Patients seek treatment at the ED to avoid waiting in long lines at the Outpatient Department (OPD). Patients perceived the assessment and investigations process in the ED to be quicker and believed that they would receive higher priority than in the OPD. Patients perceived visiting the ED could circumvent the delays incurred in visiting the OPD.

*“We came to the ED to get a prompt report of the investigation that I had to do. If I had gone to the OPD, it would have taken two to three days to get the report. They asked us to do our checkup in the OPD, but we said we would do it here in the ED as we didn’t want to wait longer to get the investigation report.”*

Pt_2, 30–40 years, female.

**Fig. 3:**
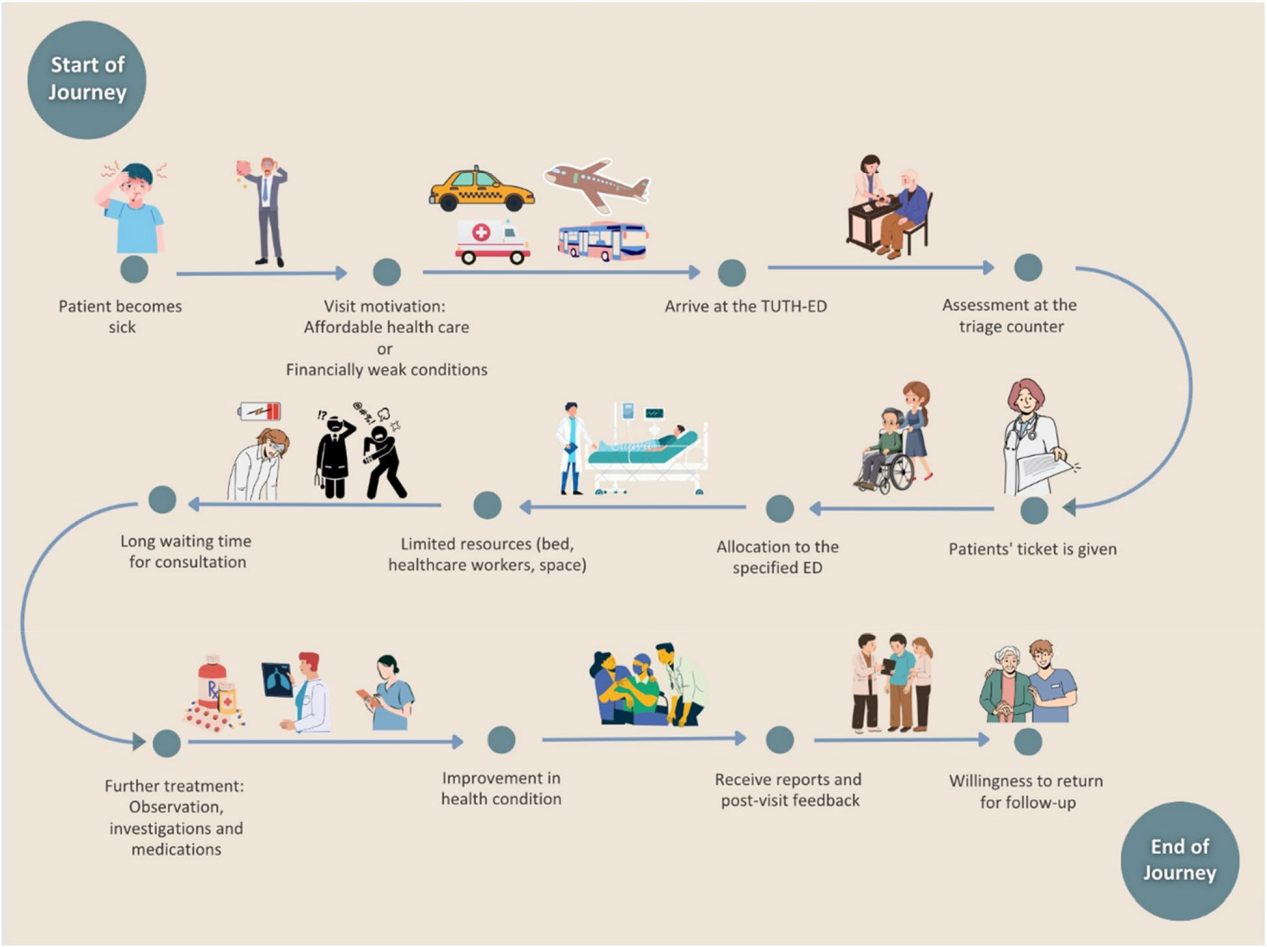
Febrile patients’ emergency department (ED) journey

The febrile patients reported various means of travel to reach the ED such as an ambulance, bus, taxi, and plane (Fig. 3). Nonetheless, a few participants mentioned that they had financial difficulty reaching the ED and had to borrow loans from formal and informal sources.

### Patients’ perspectives on services received during ED visits

Patients presenting with fever, among others, with varying levels of severity (mild, moderate, severe) received care and treatment tailored to their needs at the ED. Hence, febrile patients were satisfied with the healthcare services provided during their stay and asserted that almost all the HCWs were competent and caring towards the patients.

*“Nurses visit regularly and they are available 24 hours. The doctor treats us and prescribes the medicine; they provide us with the medicine if it is available in the room; if not, they ask us to bring it from outside.”*

Pt_1, 20–30 years, male.

*“I am satisfied with the treatment service provided. The doctors and nurses are also okay here.”*

Pt_4, 30–40 years, male.

However, some patients and caregivers observed instances of people breaking others’ turns (jumping the queue) to get the ticket at the triage and cash payment counter. According to the participants, the ED had limitations in terms of bed availability, HCWs, and space.

*“Yes, I noticed people breaking others’ turns while my brother was standing in line. I don’t know if the ones who were doing that had severe patients with them.”*

Pt_3, 50–60 years, female.

Patients also had concerns regarding poor hygiene and sanitation practices, including dirty bed sheets, and a limited number of washrooms with poor maintenance. Furthermore, there was a lack of running water in the toilet taps.

*“The ED room environment isn’t that clean. The place is very crowded and there is a lack of crowd management. There is no flush in the toilet, no mirror, there are no hand washing services available. There is no certainty when the water will come in the tap and no lock in the toilet of the ED. The toilet in the ED is very unhygienic and dirty.”*

Pt_8, 20–30 years, female.

Following treatment at the ED, the majority of patients experienced an improvement in their health conditions. Overall, the experience of febrile patients inside the ED was satisfactory, specifically in terms of the overall outcome. However, there were certain shortcomings in the services. They often had to wait for a long time to be consulted by a specialized doctor and to receive investigation reports for USG, x-ray, and molecular tests. One major concern among the febrile patients was the need to share beds with two or three other patients in the green and yellow areas.

*“I have been spending my days waiting for the doctors and the reports of the investigation. In other places, reports of different investigations come in half an hour or so in the ED. But we have to wait way longer here, maybe because this hospital is too crowded. The report of the sample that I gave in the morning took about two hours to come.”*

Pt_8, 20–30 years, female.

The patient party faced similar challenges and difficulties during their time in the ED. There were inadequate waiting areas available for both patients and the patient parties, which resulted in a distressing condition for the patient party. Furthermore, there was a lack of access to drinking water for the patients.

### Post-visit experiences

Relatively, there were fewer concerns around post-visit experience among patients and these were mostly rooted in the lack of urgency after the initial recovery. Nonetheless, most patients did express a high willingness to return to the ED of TUTH for follow-up care if needed after their discharge (Fig. 3).

“*If the doctor says anything about the follow-up, I will surely come. I won’t be facing any difficulties while coming for the follow-up.”*

Pt_1, 20–30 years, male.

## Challenges and limitations in triage practice

### Information gap

There was a notable absence of training provided to the HCWs in the ED, specifically concerning the triage system. Additionally, there was some uncertainty regarding the guidelines followed by the HCWs, with one stating adherence to WHO’s triage guidelines while three others claimed to follow Australian guidelines. This discrepancy in opinions regarding the triage guideline among the medical staff, particularly between a doctor and the nurses, highlighted the confusion and uncertainty regarding their knowledge of the process.

*“In Nepal, there are two types of triaging, one is the Australian triage scale and the other is the triaging system of WHO where the red, yellow, and green area is divided. In TUTH, the triaging is divided into red, yellow, and green areas.”*

Cl_6, 30–40 years, male.

*“For the triaging process, we have been following the Australian guidelines. We have been following the red, yellow, and green protocol for a very long time. There are no manuals or guidelines to conduct any triage process.”*

Cl_7, 40–50 years, female.

Furthermore, there was a lack of clear communication among the HCWs regarding basic procedures for patients concerning health insurance and referrals in the triage area. This created an information gap and confusion among HCWs.

In addition, the patients were not thoroughly informed about the concept of the triage process in the ED, how their treatment was prioritized, or how they were allocated to the specific ED areas. This resulted in a lack of understanding of these essential elements of emergency care.

“*I don’t know about the triage process. I have no idea about how they prioritized my treatment.”*

Pt_4, 30–40 years, male.

## Prioritization

HCWs conveyed some instances wherein some individuals would secure beds and organize admission ahead of time. This led to other patients waiting in the ED for an extended period to receive beds, despite their urgency.

*There are VIP patients like different ministers, and their treatment services should be done quickly, we cannot say we won’t do it.*

Cl_13, 30–40 years, female.

Sometimes HCWs’ family members also visited the ED and were given priority despite having relatively less severe health conditions than other patients. When HCWs’ relatives were admitted to the ED, they requested special attention from the staff. This was perceived to be inevitable, with HCWs unable to overlook the need to prioritize their family members or acquaintances. Prioritization of family members was deemed as an incentive for HCWs in return for their contribution to the hospital. While they may occasionally find it challenging to comply with regulations, HCWs did not intend to compromise the quality of care.

*“There are such cases in the ED as well, like a patient who doesn’t have to be in the red area but if it is a social case* [recommended to be prioritized by friends and relatives]*, we will allocate it in the red area. I think that might also be the reason for the ED to be managed because if I bring my parents to the ED, I would not be comfortable placing them in the crowded yellow area and my parents would also not think good of me. If there is a space in the red, I would place them there. I would find peace while doing so, they would feel relieved from the disease.”*

Cl_9, 40–50 years, female.

### Digitalization system

The ED is in the process of introducing a digital payment system. The existing manual process of recording patients’ information by the triage staff was time-consuming. Another concern was the paper quality of the emergency ticket. There were instances of misplaced tickets or visitors taking them. Thus, the ED faced challenges in keeping or maintaining triage records for longer periods.

### Healthcare worker-to-patient ratio

The available workforce in the ED was inadequate given the high throughput patient flow, which jeopardized the quality of health services. The HCW-to-patient ratio in the ED was around 1:6 or 1:8, indicating that for every HCW, there were approximately six to eight patients under their care. During periods of exceptionally high patient volume, such as during mass casualty incidents or overwhelming demand, the HCW-to-patient ratio in the ED reached 1:20.

*“The usual healthcare worker and the patient ratio is 1:8 in the ward, but the patient flow here is so high that the ratio becomes 1:20 or 1:30. That’s why I don’t feel the health resources are sufficient.”*

Cl_11, 20–30 years, female.

HCWs also shared their experience about how the quality of services was affected by the patients’ ability to attract the attention of HCWs. At the same time, patients’ ability to attract attention also jeopardized HCWs’ allocation of time based on their judgment.

*“If the visitor is clever, the patient alerts and calls the doctor and makes him/her assess the patient, but if the visitor is not clever enough, the patient may need to wait for a while.”*

Cl_7, 40–50 years, female.

### Workplace motivation

While HCWs acknowledged that the ED provided an invaluable platform for honing their skills and expertise during their formative years, there was some feedback regarding the weight of the workload, with some HCWs even considering it a potential concern. They wished their efforts to be appreciated, as they provided valuable input as HCWs while simultaneously taking into account the difficulties they confronted within the ED setting.

*“There is no motivation from the higher authority. There are only demotivating factors here. I am not saying that this hospital is not nice, I have been working here, and I have so many experiences. I have attended so many cases which I had never seen or read. But the flow of the patient is so high here I am not motivated to work here at all. It is okay during the early years; you gain all the experiences but it is physically difficult to work here. We need to stand all the time and move around all the time. We are facing workplace hazards.”*

Cl_14, 30–40 years, female.

## Discussion

The triage process in the ED at TUTH, a tertiary care hospital in Nepal was efficiently managed by skilled HCWs. The physical layout of the ED had a clear delineation of categories for care that included red, yellow, and green triage areas. This arrangement was further supported by the practice of re-triaging, a process that ensured continuous assessment of patients. However, challenges such as varying patient volumes, and constrained resources (equipment and human resources) continued to influence its effectiveness. Febrile patients were unaware of the term ‘triage’, but shared dissatisfactions related to the number of beds, space, washrooms, and overall hygiene. Patients also highlighted the limitations around the manual record and payment system which was deemed to be cumbersome.

Similar to the ED in many LMICs, the ED at TUTH had a limited number of beds which had to accommodate two to three patients, specifically in the yellow and green triage areas. The pressure to discharge patients to reduce the patient density was high, which was in part due to the low number of HCWs. This corroborates with the HCW-to-patient ratio in Nepal, which is expected to worsen as it continues to drain into Western countries [13–15]. The inadequate HCW-to-patient ratio implied that each HCW may be responsible for a large number of patients, which posed challenges in delivering timely and personalized care. In California, insufficient nurse staffing was associated with higher patient mortality rates and increased adverse events. Maintaining optimal HCW-to-patient ratios has been proven to improve patient outcomes including patient satisfaction and overall quality of the health services [16].

Emergency medical services in Nepal often face significant challenges and shortcomings similar to other LMICs [17]. Limited resources, inadequate infrastructure, and insufficient healthcare funding contribute to suboptimal emergency response systems [18]. In Sub-Saharan Africa, emergency care was found to be sub-optimal with only 16 out of 48 countries able to offer optimal emergency care services to 80% of the population who lived within a two-hour travel time from the nearest hospital [19]. The lack of well-equipped ambulances, pre-hospital care, trained personnel, and advanced medical technologies restrain the timely and effective care of patients' critical conditions [17, 20]. The shortcomings of health services were further increased during the COVID-19 pandemic where several reports attributed poor services to constraints in resources [21, 22]. In LMICs, emergency services are also indirectly affected by the referral systems which are often sinuous because of the treatment seeking landscape that is predicated by local social, and cultural values [23]. The social, political and cultural dispositions of treatment seeking behavior also pose considerable risks to vulnerable populations, leading to increased morbidity and deaths [24, 25].

While patients suffered challenges, due to the limited number of beds available, HCWs also reported significant challenges including their limitations due to inadequate human resources, a high workload, and less motivation which can consequently lead to increased turnover intentions [26]. For instance, the challenging nature of the work and long hours could contribute to the decline in motivation and ultimately affect HCWs’ functional status, impacting the triage system [27, 28]. Performance efficiency in triage can be achieved by utilizing the available resources in a timely and appropriate manner and providing a means of ensuring quality control, and staff support [29].

Workplace factors have a critical impact on HCWs’ motivation and addressing these issues is essential to ensure the delivery of high-quality healthcare. The work in the ED is fast-paced and demanding, leading to burnout and decreased motivation among staff, affecting the quality of healthcare services which can have serious consequences and affect resource allocation [30]. Low HCW motivation is associated with lower patient satisfaction, increased complaints, and higher mortality rates. Addressing motivational factors is crucial to improving workplace motivation and enhancing the quality of care [31].

Digitalization of record and payment systems and resource mobilization around it can have a significant impact. In the United States, digitization in healthcare had positive impacts on overall patient care and hospital performance [32]. The absence of digitalization in the ED obliged staff to use manual documentation, which increased the burden between both service providers and receivers. Implementing digital platforms could improve real-time reporting, analytics, and overall efficiency in patient care. A systematic review and meta-analysis showed that implementing electronic health records (EHR) systems enhances patient safety, communication among HCWs, and clinical workflow efficiency [33].

Triage is also affected by a lack of adequate information among HCWs on what triage entails, specifically the guidelines that were followed. Despite the HCWs’ lack of training regarding triage, they were still able to work efficiently. However, providing comprehensive training can greatly improve communication and collaboration among HCWs [34]. Training was found to improve the knowledge and implementation of a triage system in ED care in Iran [35]. Continued education for clinicians has been found to reinforce their practical knowledge [36]. It also helps in improving emergency processes, medical plans, and pain management [37]. There is also an information gap among patients regarding triage practices, and this is echoed in our respondents’ misunderstanding of why they did not receive priority. This further implies that there is inadequate information for patients on the triage system and thus misunderstanding of how they are prioritized. In future, HCWs should explain the rationale for triage and prioritization which can mitigate potential resentments and the workplace violence that often originates from poor communication and understanding of the medical procedures by patients and their caretakers [38, 39]. Almost 50% of HCWs working in a tertiary care hospital in Nepal have experienced workplace violence, the perpetrators being the patients and their caretakers [40].

The quality of the triage was also affected by recommendations and pressures from the authorities, specifically in prioritizing and availing beds for patients. There are concerns that such influence from the authorities might redirect resources from vulnerable patients (patients with lower economic security and underpriviledged), violating ethical principles embedded in both equity and equality in health services, and can ultimately erode the public trust in the healthcare system [41]. To foster a fair and ethical healthcare system, it is crucial to place a strong emphasis on prioritizing health equity for all [42]. Offering equitable services also applies to the interest and professional conduct of HCWs, especially when they have to balance between their priorities (e.g., when serving their relatives, colleagues, and politicians’ patients) versus any patients who attend the ED irrespective of their status or affiliations.

### Strengths and limitations of the study

This study’s emphasis on the triage procedure at a tertiary care hospital implies that the research is grounded in real-world healthcare settings. The study may have suffered from recall and social desirability biases. Nonetheless, the interviews were triangulated by the researchers’ observation notes, which may have mitigated the biases. However, the study did not follow patients until the complete cure of their illnesses, which could have offered a richer insight into the quality of care and patients’ perspectives on health services. There is an inherent lack of understanding about triage among patients, and our study explored how patients perceived the process of triage. While this study focused on the triage procedure at one tertiary care center, future studies are essential to explore how the triage system has been implemented and operates in lower-level healthcare settings, specifically in primary care.

## Conclusions

The triage at the ED of TUTH was well orchestrated by HCWs where delineated color-coded areas and re-triaging of patients facilitated efficient management of patients. The study highlighted critical gaps in current triage practices. Improving current triage management requires resource organization, including optimizing the waiting time of patients through a digitalized system.

Urgent priorities include upgrading visitor facilities, patient consultations, laboratory investigations, hygiene, and sanitation. HCWs’ recommendations to enhance the ED with more equipment, space, and beds and a dedicated triage officer to ensure 24-hour service, together with training and incentives, warrant further attention. Addressing these gaps would help to improve triage practices in emergency care at hospitals and could have wider implications for improvements in emergency care in Nepal.

### Supplementary Information

**Additional file 1.** Word document; IDI Guide; interview guides used to interview patients and HCWs during the study.

**Additional file 2.** Word document; COREQ Checklist; checklist of COREQ requirements.

## Data Availability

The datasets used and/or analysed during the current study are available from the corresponding author upon reasonable request.

## Abbreviations

COREQ: Consolidated criteria for reporting qualitative research
DM: Doctorate of medicine
ECG: Electrocardiogram
ED: Emergency department
EHR: Electronic health record
HCWs: Healthcare workers
IDI: In-depth interview
IPA: Interpretative phenomenological analysis
LMIC: Low- and middle-income countries
MO: Medical officer
OPD: Outpatient department
TUTH: Tribhuvan university teaching hospital
USG: Ultrasonography
WHO: World Health Organization
