# Supplementary material for: Triage practices for emergency care delivery: a qualitative study among febrile patients and healthcare workers in a tertiary care hospital in Nepal
Source: BMC Health Serv Res. 2024 Feb 8;24:180. doi: 10.1186/s12913-024-10663-3 (PMC10851527; doi:10.1186/s12913-024-10663-3)
Supplement: Supplementary file 1 — Additional file 1. Word document; IDI Guide; interview guides used to interview patients and HCWs during the study. [file 12913_2024_10663_MOESM1_ESM.docx]

# **Triage practices for emergency care delivery: a qualitative study among febrile patients and healthcare workers in a tertiary care hospital in Nepal**

**Additional File 1. In-depth Interview (IDI) Guide**

1. **IDI Guide for Patients**

| Semi-structured/in-depth interviews (For patients)  *(These are only guides to prompt the interviewer and interviewee to adhere to the topic. Please feel free to add the themes and concerns as you see fit)* AUDIO FILE NAME:  Introduction (read out):  I am …………………… from Tribhuvan University Teaching Hospital (TUTH) based in Kathmandu, Nepal. I am a researcher from the Institute of Medicine, Tribhuvan University, Maharajgunj, Kathmandu. The study is supported by the Foundation for Innovative New Diagnostics (FIND), a charity working to improve the identification of severe patients (triage) at the first point of contact in resource constrained settings. In this study, we are exploring how the health system (health services) operates at the emergency room (ER) of TUTH. Specifically, determining the severity of patients to make an informed decision on treatment and referral care at the first point of contact is important to save lives, particularly in settings where human resources and diagnostic tests are constrained. Accurately assessing the prognosis of patients at the ER is challenging due to the paucity of reliable information, lack of diagnostics, lack of technologies, limited health workforce, and overburdened healthcare systems, among others. Triaging is one of the critical aspects of patient management at the ER. This study aims to collate evidence on the triage process at ER that will entail exploring the triage record audit, conducting direct observation of triage practice, and examining patients’ journeys in emergency care.  In this qualitative study, we would like to know your entire experience related to your visit (pre-visit, visit, and post-visit) to the ER of TUTH. Your living experience and opinion will allow us to build robust evidence on how the triage process is operating and its impact on a patient’s diagnosis and prognosis.  This consent form is to request your voluntary participation in the study. | | | |
| --- | --- | --- | --- |
| SECTION-I: General Information | | | |
| Sociodemographic  characteristics of respondents | 1. Age |  | |
|  | 2. Gender | Male ☐ Female ☐ Other ☐ | |
|  | 3. Location | District ☐ | Village ☐ Nagarpalika ☐ Name_____________ |
|  | 4. Qualification |  | |
|  | 5. Respondent  ID |  | |
| SECTION-II: Setting description (based on the observation) | | | |
| Themes | Guide | | |

| Patient’ arrival at ER | - Is the patient walking to ER? Or is he being brought in a stretcher?   Ambulance? Any other vehicle?   - In your opinion (based on your opinion), how is the condition of the patient (sick, very sick, severe, life-threatening)? - Who attends the patient first? (triage personnel, doctor, nurse, other staff)? - Is triage being done? By who? How long was the interval between the patient’s arrival and triage? And how long did it take for the triage completion? - How did you think the triage go, and why? | |
| --- | --- | --- |
| Section-III: Pre-visit characteristics affecting treatment-seeking behaviour | | |
| Pre-visit | - Take a brief note on the patient’s illness, how it evolved, and what made them come to TUTH-ER (in contrast to others). - Who decided to come to this ER/hospital? (Household head, relatives, referral from another hospital, or anyone else) - Were there second thoughts after the decision? - Were you intending to attend other hospitals and ended up here? - How did you travel from your house to the ER? (mode of transportation: walking, vehicles, distance (km) and time) - What were the challenges in reaching out to this ER? (vehicle, financials, traffic, conflicting opinion) | |
| Section-IV: Patient’s perspective on how they received services at ER (triage and treatment) | | |
| During visit | - Who attended you first when you arrived ER? (doctor, nurse, triage personnel, other staff) - How long after you arrived at ER were you attended by someone? - Why do you think there was a delay (if any) in getting the first attention? - Have you got any idea about how they prioritized your treatment? Have you heard of screening or triage in ER? (Probe: did they see someone being prioritized over them) - If they know about the screening or triage process, explore more about what else do they know about such a process. (Explore, could they | |
|  |  | identify the indicators of triage or share an opinion on how the triage process was) |
|  | • | Did the patient or patient party notice any queue jumping? why do they think there was such queue jumping? (probe: could it be based on severity assessment? Acquaintance? Patient load? any other cause?) |
|  | • | Have you got any idea how you were allocated to this room/bed? |
|  | • | Can you explain if you have noticed how other people might have been allocated to the particular bed? Who decided and how? |
|  | • | How was the treatment or services offered to you? Are you satisfied or do you have any grievances? |
|  | • | What were the strengths of services and limitations (note in detail) |
|  | • | What could have been improved? And how? |
|  | • | Are you completely treated/cured by the services received? (Explore in detail if not) |
|  | • | What services were good and what services were not good? |
|  | • | Do you feel that you are discharged with complete recovery? (explore why for both yes and no) |
|  | • | Do you have follow-ups to attend at this hospital? Are those follow-ups mandatory or optional? |
|  | • | If you are coming from far away location, are you staying longer in Kathmandu for a follow-up visit? If so, how long? Are there any challenges in completing follow-ups? (If so, what are they?) |
| Recommendatio  ns | • | Could you tell us if there were shortcomings in services related to the initial screening/triage process? |
|  | • | Can you share, how we can improve the current screening process? |
|  | • | Do you have any recommendations or any questions I may have forgotten to ask you; could you please share? |
|  | | The End |

1. **IDI Guide for Healthcare Workers (HCWs)**

| Semi-structured/in-depth interviews (For HCWs)  *(These are only guides to prompt the interviewer and interviewee to adhere to the topic. Please feel free to add the themes and concerns as you see fit)* AUDIO FILE NAME:  Introduction (read out):  I am …………………… from Tribhuvan University Teaching Hospital (TUTH) based in Kathmandu, Nepal. I am a researcher from the Institute of Medicine, Tribhuvan University, Maharajgunj, Kathmandu. The study is supported by the Foundation for Innovative New Diagnostics (FIND), a charity working to improve the identification of severe patients (triage) at the first point of contact in resource constrained settings. In this study, we are exploring how the health system (health services) operates at the emergency room (ER) of TUTH. Specifically, determining the severity of patients to make an informed decision on treatment and referral care at the first point of contact is important to save lives, particularly in settings where human resources and diagnostic tests are constrained. Accurately assessing the prognosis of patients at the ER is challenging due to the paucity of reliable information, lack of diagnostics, lack of technologies, limited health workforce, and overburdened healthcare systems, among others. Triaging is one of the critical aspects of patient management at the ER. This study aims to collate evidence on the triage process at ER that will entail exploring the triage record audit, conducting direct observation of triage practice, and examining patients’ journeys in emergency care.  In this qualitative study, we would like to know your entire experience related to your visit (pre-visit, visit, and post-visit) to the ER of TUTH. Your living experience and opinion will allow us to build robust evidence on how the triage process is operating and its impact on a patient’s diagnosis and prognosis.  This consent form is to request your voluntary participation in the study. | | | |
| --- | --- | --- | --- |
| SECTION-I: General Information | | | |
| Sociodemographic  characteristics of respondents | 1. Age |  | |
|  | 2. Gender | Male ☐ Female ☐ Other ☐ | |
|  | 3. Workplace | Health centre ☐ | Village ☐ Name_____________ |
|  | 4. Qualification |  | |
|  | 5. Respondent  ID |  | |
| SECTION-II: Setting description (based on the observation) | | | |
| Themes | Guide | | |
| Characteristics of ER setting |  | | |

|  | - What is the size of ER (number of beds/rooms based on the observation and if confused, ask an ER physician) - What are the equipments available in ER? (make brief notes on the equipment you see, ask a physician to note them down) - Make a note (or ask) of how many health workers are on duty (doctors, nurses, paramedics, counsellors, axillary staff) - How many patients have visited in the last hour? And in a night? - How many visitors follow a patient as their accompaniers? - How is the coordination among the healthcare staff, is it concerted? Is it fragmented? - Can you explain the process or dynamics of a patient’s visit to ER? (e.g. where does he/she first go, who comes to receive him/her, and what happens next?) |
| --- | --- |
| Section-III: Resources for triage (based on observation and interview with clinicians) | |
| Resources for triage | - What resources do you have for the effective conduct of the triage process? (e.g. Guidelines for triage, training, and other resources) - Is there readily available triage guideline documents in ER? (note: where is it placed/located, what is the length of that document? how many? Also, record if anyone has read it during your observation?) - Are there training specifically targeted for a good triage process? - When was the last training conducted? Who led it? how long was it?   where did it occur? How many participants (please add as many details possible about training)   - What other triage related resources are available in ER? (Colour equipment, any other identifiers, detail as much as possible) - Are there specific personnel (human resources) for triage process? (Add details of the resource: how many, how long has he/she been recruiting, who else is he/she supported by, how does he/she function?) - Are there any back-up human resources for triage in case of mass attendance such as due to disaster, epidemics, and accidents? |

| Section-IV: Process of triage (based on observation and interview with clinicians) | |
| --- | --- |
| Process of triage | - Write your observation on how triage of a patient is being conducted? - Ask a clinician how the triage process is conducted? (Starting from receiving a patient to discharge) |
| Section-V: Challenges and opportunities for improvement in current triage process | |
| Challenges and opportunities | - What are the challenges/limitations related to the current triage process? (probe: Resources: human, material, training, motivation, managerial) - Are there challenges due to management in your organogram (your higher authorities, and your workforce)? - Is there adequate motivation among your authorities overlooking the triage process or ER (up and down the hierarchy)? - Are there policy-related barriers/challenges in operationalizing the best triage in your ER? - Is your triage process affected by internal (TUTH) and external   (national/regional/local) politics? (probe: local politics could be inherent in organogram/hierarchy, opportunities)   - Are there financial constraints in implementing a good triage process in your ER? (any non-financial ones)? - Is your triage affected by patients’ volume (numbers)? (probe: disaster, epidemics, accidents) - Can you share with me when is triage best performed? (probe: time of the day, patients’ volume) - Is triage likely to be affected during night shifts? (probe: number of human resources for triage) - If you had resources to choose, how would you change the current triage process? (probe: what resources you would add, and how would it likely change the current scenario) - What would you like to change in current triage practice? |
| Recommendatio  ns | Do you have any recommendations or any questions I may have forgotten to ask you; could you please share? |
|  | The End |
