# Supplementary material for: Triage practices for emergency care delivery: a qualitative study among febrile patients and healthcare workers in a tertiary care hospital in Nepal
Source: BMC Health Serv Res. 2024 Feb 8;24:180. doi: 10.1186/s12913-024-10663-3 (PMC10851527; doi:10.1186/s12913-024-10663-3)
Supplement: Supplementary file 2 — Additional file 2. Word document; COREQ Checklist; checklist of COREQ requirements. [file 12913_2024_10663_MOESM2_ESM.docx]

# **Triage practices for emergency care delivery: a qualitative study among febrile patients and healthcare workers in a tertiary care hospital in Nepal**

**Additional File 2. Consolidated Criteria for Reporting Qualitative Research (COREQ) checklist**

| **S.N.** | **Topic** | **Guideline Questions/description** | **Reported on page No.** |
| --- | --- | --- | --- |
| **Domain 1: Research team and reflexivity** | | | |
| *Personal characteristics* | | | |
| 1. | Interviewer/facilitator | Which author/s conducted the interview or focus group? | 5 |
| 2. | Credentials | What were the researcher’s credentials? E.g. PhD, MD | 5 |
| 3. | Occupation | What was their occupation at the time of the study? | 5 |
| 4. | Gender | Was the researcher male or female? | 5 |
| 5. | Experience and training | What experience or training did the researcher have? | 5 |
| *Relationship with participants* | | | |
| 6. | Relationship established | Was a relationship established prior to study commencement? | 6 |
| 7. | Participant knowledge of  the interviewer | What did the participants know about the researcher? e.g. personal  goals, reasons for doing the research | 6 |
| 8. | Interviewer characteristics | What characteristics were reported about the interviewer/facilitator?  e.g. Bias, assumptions, reasons and interests in the research topic | 6 |
| **Domain 2: Study Design** | | | |
| *Theoretical Framework* | | | |
| 9. | Methodological approach | What methodological approach was stated to underpin the study? | 5 |
| *Participant selection* | | | |
| 10. | Sampling | How were participants selected? e.g. purposive, convenience, consecutive, snowball | 5,6 |
| 11. | Method of approach | How were participants approached? e.g. face-to-face, telephone, email | 6 |
| 12. | Sample size | How many participants were in the study? | 5,6 |
| 13. | Non-participation | How many people refused to participate or dropped out? Reasons? | 6,7 |
| *Setting* | | | |
| 14. | Setting of data collection | Where was the data collected? e.g. home, clinic, workplace | 6,7 |
| 15. | Presence of non-participants | Was anyone else present besides the participants and researchers? | 7 |
| 16. | Description of sample | What are the important characteristics of the sample? e.g. demographic data, date | 5,6 |
| *Data collection* | | | |
| 17. | Interview guide | Were questions, prompts, guides provided by the authors? Was it pilot tested? | 5 |
| 18. | Repeat interviews | Were repeat interviews carried out? If yes, how many? | 7 |
| 19. | Audio/visual recording | Did the research use audio or visual recording to collect the data? | 6 |
| 20. | Field notes | Were field notes made during and/or after the interview or focus group? | 5 |
| 21. | Duration | What was the duration of the interviews or focus group? | 5 |
| 22. | Data saturation | Was data saturation discussed? | 6 |
| 23. | Transcripts returned | Were transcripts returned to participants for comment and/or correction? | 7 |
| **Domain 3: Analysis and Findings** | | | |
| *Data analysis* | | | |
| 24. | Number of data coders | How many data coders coded the data? | 7 |
| 25. | Description of the coding  tree | Did authors provide a description of the coding tree? | 16/Figure 2 |
| 26. | Derivation of themes | Were themes identified in advance or derived from the data? | 7 |
| 27. | Software | What software, if applicable, was used to manage the data? | 7 |
| 28. | Participant checking | Did participants provide feedback on the findings? | 7 |
| *Reporting* | | | |
| 29. | Quotations presented | Were participant quotations presented to illustrate the themes/findings? Was each quotation identified? | 12-24 |
| 30. | Data and findings consistent | Was there consistency between the data presented and the findings? | 6-19 |
| 31. | Clarity of major themes | Were major themes clearly presented in the findings? | 12-24 |
| 32. | Clarity of minor themes | Is there a description of diverse cases or discussion of minor themes? | 12-24 |
